# Cardiac troponin I as a cardiac biomarker has prognostic and predictive value for poor survival in Egyptian buffalo calves with foot-and-mouth disease

**DOI:** 10.14202/vetworld.2020.890-895

**Published:** 2020-05-14

**Authors:** Mahmoud Aly, Mohamed Nayel, Akram Salama, Emad Ghazy, Ibrahim Elshahawy

**Affiliations:** 1 Department of Animal Medicine and Infectious Diseases, Faculty of Veterinary Medicine, University of Sadat City, Egypt (Animal Medicine); 2 Department of Animal Medicine and Infectious Diseases, Faculty of Veterinary Medicine, University of Sadat City, Egypt (Infectious Diseases); 3 Department of Clinical Pathology, Faculty of Veterinary Medicine, Kafrelsheikh University, Egypt; 4 Department of Animal Medicine, Faculty of Veterinary Medicine, Alexandria University, Egypt

**Keywords:** cardiac troponin I, Egyptian buffalo calves, foot-and-mouth disease, myocarditis

## Abstract

**Background and Aim:**

Foot-and-mouth disease (FMD) causes huge economic losses in Egypt due to reductions in the production of red meat, milk, and milk by-products and can also lead to myocarditis in young animals. The aim of our study was to evaluate cardiac biomarkers, in particular cardiac troponin I (cTnI), and to reveal the relations of cardiac biomarkers with poor survival in FMD-infected Egyptian buffalo calves.

**Materials and Methods:**

Forty-two Egyptian buffalo calves were included in this study. The calves were divided into 12 apparently healthy control calves and 30 calves clinically diagnosed with FMD during a disease outbreak in Menofia and Behera Governorates, Egypt. The diseased calves were divided, according to age, into 13 calves <3 months old and 17 calves between 3 and 6 months old. The animals were examined clinically and subjected to analysis of cardiac biomarkers.

**Results:**

Biochemical analysis revealed significant elevations of cardiac biomarkers, especially creatine kinase myocardial band (CK-MB), lactate dehydrogenase (LDH), alanine aminotransferase (ALT), aspartate aminotransferase (AST), cardiac troponin T (cTnT), and cardiac troponin I (cTnI) in FMD-infected calves in comparison with control calves. There was a significant association between cTnI and poor survival in infected calves.

**Conclusion:**

Cardiac biomarkers could be used as a rapid method for diagnosis of myocarditis induced by FMD in Egyptian buffalo calves. In addition, cTnI is a very sensitive and accurate tool for determining myocardial cell damage in the earlier stages of the disease and a good predictor of poor survival in calves.

## Introduction

Foot-and-mouth disease (FMD) is a highly contagious vesicular disease affecting cloven-hoofed domestic and wild animals that are characterized by vesicles in the mouth and on the feet [1]. FMD is caused by foot-and-mouth disease virus (FMDV) belonging to the genus *Aphthovirus* of the Picornaviridae family. Seven serotypes have been distinguished: O, A, C, Asia 1, South African Territories (SAT) 1, SAT 2, and SAT 3 [2]. FMD is one of the most economically important livestock diseases [3]. In calves, it is characterized by high mortality. Acute severe myocardial injury is the main cause of death in most young calves, without the appearance of the characteristic blister lesions seen in adult cattle [4]. Mortality from FMD is low (5%) in adult cattle, but it is higher (20% or more) in calves due to myocardial injury [5]. Acute myocarditis in calves is characterized histopathologically by hyaline degeneration, necrosis of muscle fibers, and intense infiltration, mainly of lymphocytes [4].

Clinical diagnosis of heart disorders in cattle, especially myocarditis, remains challenging because it depends mainly on physical examination, cardiac auscultation, and sudden death in the field [6]. However, there are some biochemical markers for the diagnosis of myocardial injury, including lactate dehydrogenase (LDH), aspartate aminotransferase (AST), and creatine kinase myocardial band (CK-MB) [7].

The best cardiac biomarkers of myocardial injury are the cardiac troponins, especially cardiac troponin I (cTnI). cTnI is a subunit of the troponin complex that binds to tropomyosin and actin on the thin filaments of striated muscle fibers [8]. cTnI is the only troponin expressed in the myocardium [9]. The serum level of cTnI increases after acute myocardial injury because of leakage from destroyed myocardial cells [10,11]. Therefore, Cardiac biomarkers could be important prognostic tools in FMD-infected calves.

The current study aimed to investigate the clinical value of cardiac biomarkers for the prognosis and diagnosis of FMD in Egyptian buffalo calves and to determine the correlation of cTnI with poor survival in diseased calves.

## Materials and Methods

### Ethical approval

This study was approved by the Institutional Animal Ethics Committee, Faculty of Veterinary Medicine, University of Sadat City.

### Animals

Forty-two Egyptian buffalo calves were included in the study. The calves were divided into 12 apparently healthy control calves and 30 calves clinically diagnosed with FMD infection during a disease outbreak in Menofia and Behera Governorates, Egypt. The diseased calves were divided into 13 calves <3 months old and 17 calves aged 3-6 months. The calves were examined clinically for pulse rate, temperature, respiratory rate, and ruminal movement and by auscultation to detect heart sounds and arrhythmias, according to the methods of Smith [12].

### Blood samples

Ten milliliters of blood was taken from the calves by jugular vein puncture, and about 3 mL of blood was transferred into vacuum ethylenediaminetetraacetic acid (EDTA)-coated tubes for viral isolation. The remaining amount of blood was divided into two parts, one in heparinized tubes for biochemical analysis and the other into tubes without anticoagulant for serum isolation. Serum samples were harvested by centrifugation at 3000 rpm for 10 min and preserved at −20°C until analysis.

### Biochemical analysis

The activities of CK-MB, LDH, AST, and alanine aminotransferase (ALT) were estimated spectrophotometrically by standard procedures using commercial kits (BioMérieux, Egypt). The level of cardiac troponin T (cTnT) was quantitatively determined using electrochemiluminescence technology third-generation cTnT developed by Roche Diagnostics, Indianapolis, GMBH, Germany. The concentration of cTnI was determined in samples of serum using Card-I-kit Combo Test (AboaTech, Turku, Finland). The test was carried out according to the manufacturer’s instructions.

Acute-phase proteins (APPs), including serum amyloid A (SAA), serum haptoglobin (Hp), and C-reactive protein (CRP), were measured with a commercially available bovine ELISA kit (Life Diagnostics, West Chester, PA, USA), according to the manufacturer’s instructions.

### Isolation and identification of the virus

Samples were obtained from apparently healthy calves and diseased calves for disease confirmation and serotyping. The samples were analyzed by the conventional reverse-transcriptase polymerase chain reaction (RT-PCR) method. Total RNA was extracted from EDTA-mixed whole blood using TRIzol Reagent (TRIzol™ Plus RNA Purification Kit; catalog number 12183555) following the manufacturer’s instructions. After RNA was extracted from the blood, RT-PCR was performed for serotype identification of FMDV using the specific primers described in Table-1[13-15]. For the detection of FMDV, the RNA from 20 samples was subjected to RT-PCR by TIANGEN, Quant One-Step RT-PCR Kit; catalog number KR113, according to the manufacturer’s instructions. Each One-Step RT-PCR mixture tube (50 µL) contained a different serotype primer for each separate reaction. The pan-serotype RT-PCR cycling protocol was similar, except that the annealing temperature was modified to 55°C with UTR RT-PCR. Thermocycler G-Storm was used for the detection of FMDV by RT-PCR. The PCR products were analyzed by electrophoresis through 1.5% agarose gel containing ethidium bromide (0.5 μg/mL), and the image of the amplified product was captured using a gel documentation system (Biospectrum UVP, UK).

**Table-1:** Sequences of the primers used for conventional reverse transcriptase polymerase chain reaction.

FMDV serotype	Sequence (5-3)	Size (bp)	References
Pan serotypes (5’ UTR)	F: GCC TGG TCT TTC CAG GTC T R: CCA GTC CCC TTC TCA GAT C	328	[13]
O	R: CATGTCYTCYTGCATCTGGTT F: AGATTTGTGAAAGTDACACCA	658	[15]
A	R: CATGTCYTCYTGCATCTGGTT F: CTTGCACTCCCTTACACCGCG	427	[15]
SAT2	R: GAAGGGCCCAGGGTTGGACTC F: CACTGCTACCACTCR GAG TG	880	[14]

### Statistical analysis

All data were represented as means±standard error of the mean. The groups were compared by the independent samples t-test. Time-to-event and survival variables were analyzed by the Kaplan–Meier test in the different groups which were tested using the log-rank test. Linear correlations between clinical parameters and serum biomarkers were determined by Spearman’s rank correlation test. p<0.05 was considered to indicate statistical significance. Statistical analyses were performed using SPSS version 23 (IBM, Armonk, NY, USA), and graphs were created with Prism 5 (GraphPad, La Jolla, CA, USA).

## Results

### Observed clinical signs in FMD-infected Egyptian buffalo calves

**Figure-1:**
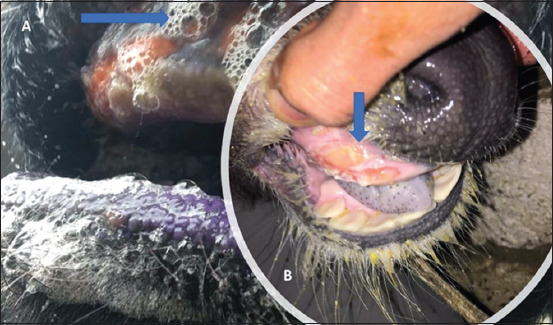
The clinical signs of Foot-and-mouth disease-infected Egyptian buffalo calves showed (A) Salivation (B) vesicular ulceration of dental pad.

The clinical findings in FMD-infected Egyptian buffalo calves were mainly vesicular lesions on the feet and/or mouth, smacking of the lips and salivation (Figure-1), anorexia, dullness, and refusal to suckle. There were adult buffaloes showing clinical symptoms of FMD at the same farm with these calves.

Heart rate, respiratory rate, and internal body temperature were significantly higher in calves with FMD than in healthy controls (p<0.05), and ruminal motility was significantly lower in calves with FMD than in healthy controls (p<0.05) (Table-2). On close examination of the heart by auscultation, we detected the frictional, muffled, and tinkling-splashing sounds of myocarditis and pericarditis in different calves, especially those under 3 months of age (two calves with frictional, four with muffled, and five with tinkling-splashing sounds). In calves 3-6 months of age, but not in the whole group of infected calves, we observed three calves with non-abnormal heart sounds, five with frictional sounds, four with muffled sounds, and five with tinkling-splashing sounds.

**Table-2:** Clinical findings in control and FMD diseased calves.

Variable	Apparently healthy calves	Diseased calves	
	
	No. (12)	Calves <3 months. No. (13)	Calves >3-6 months. No. (17)
Oral lesion	-	+(6)	+(9)
Foot lesion	-	+(2)	+(4)
Both oral and foot lesion	-	+(5)	+(4)
Myocarditis signs	-	+(11)	+(14)
Non-abnormal sounds	12	+(0)	+(3)
Frictional		+(2)	+(5)
Muffled		+(4)	+(5)
Tinkling splashing		+(5)	+(4)
Rectal temperature (°C)	38.5±0.5	41.8±0.6*	40.7±0.3*
Respiration rate “breathe/minute”	31.2±0.25	65.5±0.24*	45.9±0.54*
Heart rate	76.5±1.56	120.5±1.88*	110.5±1.87*
Ruminal motility/2 min	3.3±0.1	1.1±0.2*	0.5±0.01*

(Means±SD)

* p<0.05, **p<0.01 and ***p<0.001. FMD=Foot-and-mouth disease

### Molecular detection of FMDV

**Figure-2:**
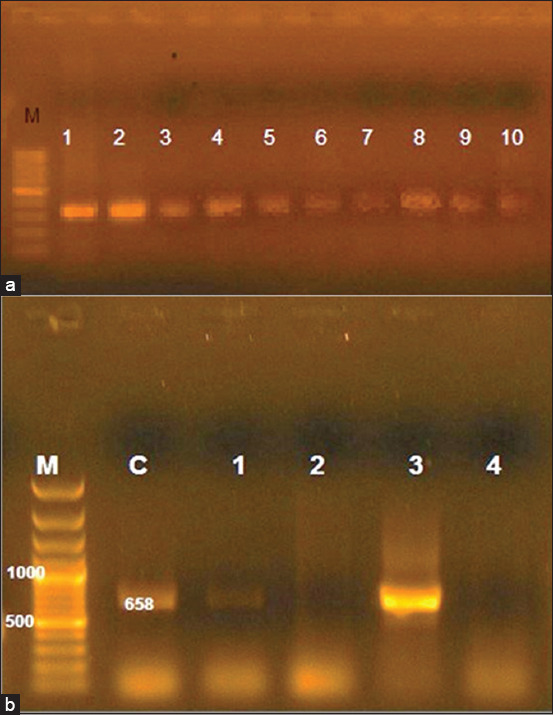
(a) Reverse-transcriptase polymerase chain reaction (RT-PCR) targeting pan serotypes (328 bp). Lane 1: Control positive, lane 2-10: Clinical samples, M: Molecular weight marker. (b) RT-PCR targeting O-serotype (658 bp). Lane C: Control positive, lane 1-4: Clinical samples, M: Molecular weight marker.

The primary results of the tests of 20 representative RNA samples collected from calves with clinical FMD using conventional RT-PCR targeting the 5’ UTR region and using the pan-serotypic primers targeting independently conserved regions of the FMDV genome showed that there was initial evidence for FMDV, as specific bands were detected at 328 bp (Figure-2a). Using serotype-specific primers of the three virus serotypes circulating in Egypt (O, A, and SAT 2), conventional RT-PCR detected O serotype-specific bands at 658 bp (Figure-2b). Serotypes A and SAT 2 were not detected.

### Cardiac biomarkers and some APPs in apparently healthy and FMD-infected Egyptian buffalo calves

Comparison of data obtained from different groups of calves (control, diseased under 3 months old, and diseased between 3 and 6 months old) revealed a moderate elevation in CK-MB levels (p<0.05) in diseased compared with control calves, with no significant difference between younger and older diseased calves. The mean levels of serum LDH, AST, and cTnT were significantly higher (p<0.01) in diseased calves than in control calves, with no significant differences between younger and older diseased calves. There were no significant differences between groups (control, diseased under 3 months old, and diseased between 3 and 6 months old) in ALT levels.

cTnI levels showed a highly significant elevation (p<0.001) in diseased FMD calves compared with controls. cTnI levels in diseased calves under 3 months old were significantly elevated compared with levels in diseased calves between 3 and 6 months old (Table-3).

**Table-3:** Cardiac biomarkers in healthy and FMD diseased Egyptian buffalo calves.

Parameter	Apparently healthy calves	Diseased calves	

		Calves <3 months	Calves >3-6 months
CK-MB (U/L)	125.64±0.87	201.51±0.73*	189.71±0.93*
LDH (U/L)	505.75±0.77	750.73±0.65**	733.53±0.65**
AST (U/L)	78.04±0.54	490.50±0.69**	410.50±0.57**
ALT (U/L)	48.03±0.34	65.03±0.1	58.03±0.7
cTnT (ng/mL)	0.081±0.02	0.98±0.01**	0.93±0.03**
cTnI (ng/mL)	0.035±0.002	2.89±0.23***	2.19±0.02**

(Means±SD)

* p<0.05,

** p<0.01, and

*** p<0.001. FMD=Foot-and-mouth disease, CK-MB: Creatine kinase myocardial band, LDH: Lactate dehydrogenase, ALT: Alanine aminotransferase, AST: Aspartate aminotransferase, cTnT: Cardiac troponin T, cTnI: Cardiac troponin I

APPs, including Hp, SAA, and CRP, showed highly significant elevations (p<0.05) in diseased calves compared with control calves, with no significant differences between younger and older diseased calves (Table-4).

**Table-4:** Acute-phase proteins in healthy and FMD diseased Egyptian buffalo calves.

Parameter	Apparently healthy calves	Diseased calves	

		Calves <3 months	Calves >3-6 months
Haptoglobin (mg/l)	0.05±0.001	1.56±0.11*	1.36±0.11*
Serum amyloid A (mg/l)	5.56±0.03	39.46±0.31*	36.7±0.81*
C-reactive protein (μg/mL)	39.03±0.41	97.05±0.01*	87.34±0.01*

(Means±SD)

* p<0.05, **p<0.01, and ***p<0.001. FMD=Foot-and-mouth disease

### Cardiac biomarkers and some APPs in surviving and non-surviving FMD-infected Egyptian buffalo calves

Mortality was reported in both younger and older FMD-infected calves. Seven calves <3 months old and five calves 3-6 months old died. Analysis of the cardiac biomarkers and APPs of the 18 survivors and 12 non-survivors revealed a highly significant increase in cTnI levels in non-survivors compared with survivors (p<0.001). There were moderately significant increases in LDH, AST, and cTnT levels in non-survivors compared with survivors (p<0.01), as well as a mild elevation in CK-MB level (p<0.05) in non-survivors compared with survivors. There were no differences in ALT levels between nine non-survivors and six survivors (Table-5).

**Table-5:** Cardiac biomarkers and some acute-phase proteins in survivors and non-survivors FMD diseased Egyptian buffalo calves.

Variables	Survivors (n=6)	Non-survivors (n=9)
CK-MB (U/L)	165.64±0.45	245.51±0.43*
LDH (U/L)	556.75±0.56	770.73±0.61**
AST (U/L)	290.04±0.54	499.40±0.5**
ALT (U/L)	52.03±0.34	69.03±0.1
cTnT (ng/mL)	0.51±0.02	1.03±0.01**
cTnI (ng/mL)	1.45±0.003	3.43±0.21***
Haptoglobin (mg/l)	0.89±0.02	1.88±0.11*
Serum amyloid A (mg/l)	25.56±0.03	42.46±0.31*
C-reactive protein (μg/mL)	67.03±0.41	99.05±0.01*

* p<0.05,

** p<0.01, and

*** p<0.001. FMD=Foot-and-mouth disease, CK-MB: Creatine kinase myocardial band, LDH: Lactate dehydrogenase, ALT: Alanine aminotransferase, AST: Aspartate aminotransferase, cTnT: Cardiac troponin T, cTnI: Cardiac troponin I

### Association of cTnI levels with poor survival in FMD-infected Egyptian buffalo calves

**Figure-3:**
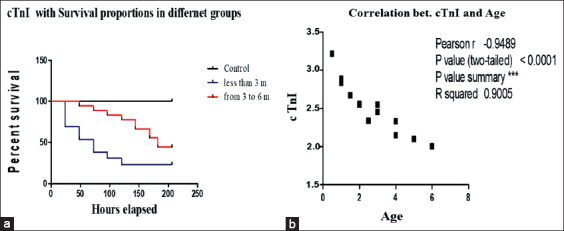
(a) The survival percent of both diseased subgroups (<3 months and from 3 to 6 months). (b) The inverse correlation curve between cTnI and age of the calves.

Calves with cTnI serum levels >3.2 ng/mL were 3.5 times more likely to die than calves with normal levels (95% CI, 1.4-20.8; p=0.01). There was a strong inverse correlation between cTnI level and age of the calf (Figure-3).

## Discussion

FMD is a highly contagious disease that can lead to myocarditis in young animals. Acute severe myocardial injury may result in death, especially in neonatal calves [4]. We, therefore, investigated the main clinical manifestations and cardiac biomarkers of FMD-infected Egyptian buffalo calves to determine the correlations between cTnI and poor survival in diseased calves.

The clinical findings in FMD-infected calves were mainly vesicular lesions on the feet and/or mouth, smacking of the lips and salivation, anorexia, dullness, and refusal to suckle from the dam. These signs were also recorded by Constable *et al*. [16]. The clinical picture in FMD-infected calves includes increases in pulse rate, respiration rate, and body temperature and a decrease in ruminal motility. Close auscultation of the heart detected the frictional, muffled, and tinkling-splashing sounds of myocarditis and pericarditis, especially in calves <3 months old, which were also reported in suckling calves by Karapinar *et al*. [17].

It has been reported that the use of some biomarkers for the detection of heart damage is very important for the diagnosis of myocarditis. Therefore, we focused on measurements of CK-MB, LDH, ALT, AST, cTnT, and cTnI.

We observed a moderate elevation in CK-MB levels (p<0.05) in FMD-infected calves. This can be used as an indicator of myocarditis and damage to myocardial cells. CK-MB is a subunit of CK that increases in the blood if the heart muscle is damaged [18-20]. In humans, serum CK-MB levels peak 24 h after the beginning of acute cardiac injury and are within the normal range during the very early stages of myocarditis [5].

Mean serum LDH levels were highly significantly increased (p<0.01) in FMD-infected calves compared with control calves, in agreement with the previous findings [5,21]. LDH and AST levels are elevated after injury to the liver, skeletal muscle, or cardiac muscle [18]. Therefore, higher levels of LDH and AST were caused by cardiac muscle damage.

The highly significant elevations in cTnI (p<0.001) and cTnT (p<0.01) levels in FMD-infected calves compared with control calves indicate myocardial cell injury and degeneration. This finding is supported by the work of Sobhy *et al*. [19] and Dawood and Alsaad [21] and is related to the myotropism effect of FMDV, especially to heart tissue, which leads to myocardial damage and cardiac insufficiency [22]. In our study, cTnI levels were significantly higher in FMD-infected calves <3 months old than in calves between 3 and 6 months old. Another study showed that the risk of myocarditis in FMD infection was directly related to age and was higher in calves under 2 months old [5].

The association of cTnI levels with poor survival in FMD-infected calves was confirmed by the significant increase (p<0.001) in cTnI levels in non-surviving animals compared with survivors. Cardiac arrhythmias, rather than myocarditis, may be responsible for the elevated levels of cTnI, as a relationship between cardiac arrhythmias and elevated levels of cTnI has been reported in humans [23]. There may be additional causes of increased cTnI levels; 100% of non-surviving calves in our study, but none of the survivors, had cardiac arrhythmias.

We found significant increases in the levels of the APPs HP, SAA, and CRP in FMD-infected calves compared with healthy controls, indicating a rapid acute-phase response that coincided with the appearance of clinical signs of disease. Both HP and SAA had significantly increased serum levels in FMD-infected calves [24]. APPs act as the immediate host defense against inflammation, injury, and infection by enclosing microorganisms and their by-products, activating the complement system, scavenging free hemoglobin and radicals, and modulating the host’s immune response [25].

## Conclusion

Cardiac biomarkers revealed the existence of myocarditis and its degree in FMD-infected calves. In addition, cTnI is a very sensitive and accurate tool for determining myocardial cell damage in the earlier stages of the disease and a good predictor of poor survival in calves.

## Authors’ Contributions

MA: Designed the study. MA, IE, MN, AS, and EG: Methodology. MA, IE, MN, and AS: Validation. MA, IE, MN, and AS: Formal analysis. MA, IE, MN, and AS: Investigation. MA, IE, MN, and AS: Data curation. MA: Drafted the manuscript. MA, IE, MN, AS, and EG: Visualization. All authors have drafted, revised and approved the final manuscript.
